# Marine Cold Seep ANME‐2/SRB Consortia Produce Their Lipid Biomass From Inorganic Carbon

**DOI:** 10.1111/1462-2920.70213

**Published:** 2025-12-01

**Authors:** Lennart Stock, Gunter Wegener, Yueqing Wang, Yannick Zander, Marcus Elvert

**Affiliations:** ^1^ MARUM‐Center for Marine Environmental Sciences University of Bremen Bremen Germany; ^2^ Faculty of Geosciences University of Bremen Bremen Germany; ^3^ Alfred Wegener Institute for Polar and Marine Research Bremerhaven Germany; ^4^ Max Planck Institute for Marine Microbiology Bremen Germany

**Keywords:** ANME‐2/SRB, AOM consortia, carbon assimilation, cold seeps, lipid biomarkers, membrane modulation, SIP

## Abstract

In cold seeps, anaerobic methanotrophic archaea (ANME) and sulphate‐reducing bacteria (SRB) oxidise methane to inorganic carbon (IC) coupled to sulphate reduction. While catabolic pathways are well resolved, carbon flow into biomass as well as the functional roles of lipid biomarkers remain unclear. We conducted lipid stable isotope probing (lipid‐SIP) experiments with Astoria Canyon sediments dominated by ANME‐2/SRB consortia and incubated samples with either ^13^C‐labelled methane (^13^CH_4_) or dissolved IC (DI^13^C). Lipid‐specific *δ* ^13^C analysis showed higher ^13^C incorporation from DI^13^C than from ^13^CH_4_. After 30 days, *δ* ^13^C values were up to +417‰ in SRB‐specific fatty acids (e.g., C_16:1ω5c_, cyC_17:0ω5,6_) and +126‰ in ANME‐2‐specific isoprenoid lipids (e.g., archaeol, crocetane). Based on these values, we calculated carbon assimilation rates and found that both partners primarily assimilate IC. Remarkably, IC assimilation in SRB lipids was eight times higher than in ANME lipids, suggesting that ANME may use additional yet‐to‐be‐identified carbon sources, potentially produced by their partner SRB. By examining the stepwise ^13^C‐enrichment of ANME‐ and SRB‐derived lipids, we further delineate biosynthetic pathways for archaeal and bacterial diether lipid formation and highlight crocetane as a bilayer‐modulating isoprenoid hydrocarbon potentially affecting membrane fluidity and proton permeability.

## Graphical abstract

Lipid‐stable isotope probing (SIP) experiments in Astoria Canyon sediments revealed that both ANME‐2 and SRB primarily assimilate dissolved inorganic carbon (DIC or HCO_3_^‐^), not methane, into biomass. SRB‐specific lipids showed eightfold higher DI^13^C‐assimilation than ANME lipids, suggesting SRB directly assimilate DIC, while ANME assimilate an alternative carbon source, potentially provided by SRBs.

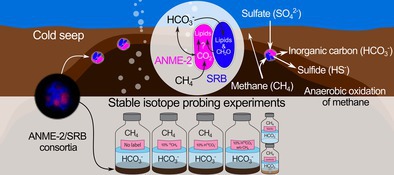

## 1 Introduction

The anaerobic oxidation of methane (AOM) with sulphate as electron acceptor is a major methane sink, consuming approximately 90% of the methane produced in marine sediments (Hinrichs and Boetius 2002; Knittel and Boetius 2009; Reeburgh 2007). AOM is mediated by syntrophic consortia composed of anaerobic methanotrophic archaea (ANME), which oxidize methane to inorganic carbon (IC), and sulphate‐reducing bacteria (SRB) that use reducing equivalents released during AOM to reduce sulphate to sulphide (Orphan et al. 2001). The overall redox reaction for AOM coupled to sulphate reduction (SR) can be summarized as CH_4_ + SO_4_^2−^ → HCO_3_^−^ + HS^−^ + H_2_O, with a Δ*G* at under in situ conditions of −20 to −40 kJ mol^−1^ (Knittel and Boetius 2009; Larowe et al. 2008; Thauer 2011).

ANME archaea comprise three polyphyletic lineages within the phylum Halobacteriota. ANME‐1 (*Ca*. Methanophagales) forms a distinct order dominating deep sulphate methane transition zones (SMTZs), hydrothermal and hypersaline environments (Knittel et al. 2019; Ruff et al. 2015), and microbial mats (Michaelis et al. 2002). ANME‐3 (*Ca*. Methanovorans) is a genus within the family Methanosarcinacea, and is closely related to Methanococcoides. This uncultured ANME group is often found at mud volcanoes (Lazar et al. 2011; Niemann et al. 2006). ANME‐2 dominates most cold seep environments, especially those with high AOM activity (e.g., Niemann et al. 2006; Omoregie et al. 2008; Treude et al. 2003). This lineage includes several subgroups, including ANME‐2a, ‐2b (*Ca*. Methanocomedens, *Ca*. Methanomarinus), ‐2c (*Ca*. Methanogaster) (Chadwick et al. 2021; Knittel and Boetius 2009; Wegener et al. 2022) and the terrestrial ANME‐2d clade (*Ca*. Methanoperedens, Haroon et al. 2013).

Metagenomic and metatranscriptomic studies revealed that all ANME lineages oxidise methane via a C_1_‐pathway resembling reverse methanogenesis (Chadwick et al. 2022; Hallam et al. 2004; Meyerdierks et al. 2010; Thauer 2011; Wang et al. 2014). Most marine ANME grow in syntrophy with SRB of the Desulfobacterota phylum, as demonstrated by fluorescence in situ hybridization (FISH, e.g., Boetius et al. 2000; Metcalfe et al. 2021; Orphan et al. 2001; Osorio‐Rodriguez et al. 2023). In cold seep environments, SRB typically belong to the Desulfococcus/Desulfosarcina (DSS) clade (e.g., Boetius et al. 2000; Orphan et al. 2001). They grow on the reducing equivalents from ANME and, utilise these for sulphate reduction (McGlynn et al. 2015; Wegener et al. 2015). Recent studies suggest that the consortia members perform direct interspecies electron transfer mediated by cytochrome‐based nanowires (Chadwick et al. 2022; McGlynn et al. 2015; Wegener et al. 2015). Metagenomic analysis indicates that most SRB encode the Wood–Ljungdahl pathway, suggesting that the partner bacteria are autotrophs (Hügler and Sievert 2011; Skennerton et al. 2017). Similarly, ANME encode an often slightly modified archaeal version of this pathway (Chadwick et al. 2022). However, in ANME the C1‐branch of its Wood–Ljungdahl pathway is central to reverse methanogenesis pathways, which enable the oxidation of methane to CO_2_ (Thauer 2011).

Both, ANME and the SRB partner produce characteristic ^13^C‐depleted membrane lipids, indicating the incorporation of methane‐derived carbon. ANME‐2 archaea typically produce the glycerol dialkyl diethers sn‐2 hydroxyarchaeol (sn2‐OH‐archaeol) and archaeol, while ANME‐1 archaea produce mainly glycerol dialkyl glycerol tetraethers (GDGTs), all with *δ* ^13^C values ranging from −70‰ to −130‰ relative to Vienna Pee Dee Belemnite (VPDB) (Elvert et al. 2005; Hinrichs et al. 2000; Niemann and Elvert 2008; Orphan et al. 2002; Blumenberg et al. 2004). These are among the most ^13^C‐depleted biogenic compounds known with *δ* ^13^C values much lower than those of photo‐ and lithoautotrophic microorganisms, with, for example, phototrophic algae producing lipids and biomass with *δ* ^13^C values between −15‰ and −35‰ (Goericke and Fry 1994; Henderson et al. 2024). The dominant SRB lipids in AOM consortia include fatty acids such as C_16:1ω5c_ and cyC_17:0ω5,6_, with *δ* ^13^C values between −60‰ and −100‰ (e.g., Blumenberg et al. 2004; Elvert et al. 2003). The variability in lipid *δ* ^13^C values of ANME and SRB reflects the wide carbon isotope range of environmental methane (−20‰ to −110‰), depending on whether it is of thermogenic or biogenic origin (Claypool and Kaplan 1974; Whiticar 1999). Because AOM generates ^13^C‐depleted dissolved inorganic carbon (DIC), the question remains whether consortia members assimilate methane, DIC, both, or additional carbon sources.

To resolve this question, multiple studies have repeatedly applied lipid stable isotope probing (lipid‐SIP; Boschker and Middelburg 2002; Wegener, Kellermann, and Elvert 2016) to track the assimilation of ^13^C‐labelled carbon substrates into microbial lipids of AOM‐active cold seep sediments, microbial mats, and enrichment cultures (Bertram et al. 2013; Blumenberg et al. 2005; Jagersma et al. 2009; Kellermann et al. 2012; Wegener, Krukenberg, et al. 2016; Wegener et al. 2012, 2008). However, this often led to different conclusions. Early studies used only ^13^CH_4_ methane (Blumenberg et al. 2005; Jagersma et al. 2009), whereas subsequent work also tested DI^13^C as potential carbon substrate (Bertram et al. 2013; Kellermann et al. 2012; Wegener, Krukenberg, et al. 2016; Wegener et al. 2012, 2008). ANME‐2d archaea, which perform nitrate‐dependent AOM, form biomass from both, methane and IC, suggesting a mixotrophic growth (Kurth et al. 2019). In contrast, ANME‐1 archaea appear to preferentially assimilate IC, indicating chemoautotrophy as the dominant mode (Kellermann et al. 2012; Treude et al. 2007; Wegener, Krukenberg, et al. 2016). In the case of ANME‐2, however, studies indicate preferential assimilation of IC rather than methane (Bertram et al. 2013; Wegener, Krukenberg, et al. 2016). However, so far, the relative contributions of methane and inorganic carbon as biomass carbon sources are not sufficiently established (Wegener et al. 2008).

To address this issue, we conducted lipid‐SIP experiments with cold seep sediments from the Astoria Canyon off the Oregon coast, which are dominated by ANME‐2/SRB consortia. Under AOM‐favorable conditions, we incubated the samples with DI^13^C, with and without CH_4_, as well as with ^13^CH_4_ and DIC. This design allowed us to quantify AOM‐dependent carbon assimilation and lipid turnover from either IC or methane. Our results provide new insights into the metabolic strategies of ANME‐2, in particular its ability to produce its biomass from IC. In addition, we tracked the time course of ^13^C‐assimilation into archaeal and bacterial lipids, which sheds light on lipid biosynthetic pathways and supports the hypothesis that archaea synthesize isoprenoid hydrocarbons as membrane lipid modulators.

## 2 Material and Methods

### 2.1 Site Description and Sample Collection

The Astoria Canyon, located on the northern Cascadia margin, hosts numerous cold seep sites, reflecting the widespread occurrence of such features across the entire Cascadian margin (Baumberger et al. 2018; Merle et al. 2021). During the R/V Atlantis cruise AT50‐14 in August 2023, we collected sediment cores from the cold seep site “H1867” in Astoria Canyon, Oregon, USA (lat.: 46.240784° N, long.: 124.602954° W, water depth: 760 m). Geochemical characterisation of the same cold seep system can be freely accessed through the Biological and Chemical Oceanography Data Management Office (BCO‐DMO) repository (www.bco‐dmo.org/dataset/959765) (Lalk et al. 2025). The location was previously found during E/V Nautilus cruise NA128 with the ROV Hercules (Dive H1867; Wishnak 2022). The first 20 cm of the push core were pooled in glass bottles and kept under anoxic conditions until further preparation of the experiment in the laboratory.

### 2.2 Incubation of Sediment Slurries

A sediment slurry was prepared with 200 g of cold seep sediment and 500 mL of artificial anoxic seawater medium (Laso‐Pérez et al. 2018) with 25 mM magnesium sulphate and 30 mM sodium bicarbonate (i.e., DIC; Merck). These slurries were then evenly distributed into six 250‐mL serum vials and filled up to 100 mL with the anoxic medium. The vials were incubated with CH_4_ as the energy source. Rates of methane‐dependent sulphide production were monitored using an established colorimetric assay (Cord‐Ruwisch 1985; Laso‐Pérez et al. 2018). All samples showed instant sulphide production (> 5 mM in 30 days). For the SIP experiment, all slurries were pooled and supplied with new medium with 27 mM DIC. This slurry was distributed in 13,250‐mL serum vials. Each bottle received 50 mL of sediment slurry and 100 mL of anaerobic seawater medium, leaving 100 mL of gas headspace. Each vial was spiked with either ^13^C‐labelled or non‐labelled methane or DIC to achieve the following experimental conditions (see Table 1 for details): (1) 30 mM DIC and ~30 mM CH_4_, (2) 10% labelled 30 mM DIC and ~30 mM CH_4_, (3) 30 mM DIC and 10% labelled ~30 mM CH_4_ and (4) 30 mM DIC and no CH_4_. We aimed for 30 mM concentrations and a labelling strength of 10% ^13^C for both DIC and CH_4_ and received ~5000‰ DIC and ~10,000‰ CH_4_ isotope values (Table 1). In addition, blank incubations with about 5 g of combusted sea sand were prepared for the treatments (1), (2) and (3), while killed controls, using the original sediments, were set up for the treatments (2) and (3). For the latter two, additions of 10 mL of 20% zinc chloride (Merck) solution were used. Treatments containing labelled DIC received 3 mM ≥ 99% of DI^13^C (Sigma‐Aldrich), while non‐labelled treatments received 3 mM non‐labelled DIC to compensate for the missing 3 mM. All treatments containing CH_4_ received 1 bar of CH_4_/CO_2_ (90/10%, v/v) (Air Liquide). Treatments containing labelled CH_4_ received 10 mL of ≥ 99% ^13^CH_4_ (Sigma‐Aldrich). For treatments without CH_4_, a gas mixture of N_2_/CO_2_ (90/10%, v/v; Air Liquide) of 1 bar was used.

**TABLE 1:** Overview of carbon isotopic compositions, concentrations and rates of geochemical data of all individual SIP incubation experiments.

Treatment	Time	CH_4_	DIC	DIC	SO_4_^2−^	H_2_S	H_2_S	SO_4_^2−^	DIC	CH_4_
	[days]	*δ* ^13^C [‰]		Conc. [mM]			Rates [μmol g_dw_^−1^ d^−1^]			
DIC w/ CH_4_	0	−43	−11	32	25	0.2	NA	NA	NA	NA
DIC w/ ^13^CH_4_	10	~9000	205	36	24	1.4	3.5	−7.1	5.8	2.5
	20	~15,000	918	39	22	3.3	4.8	−4.8	9.5	4.0
	30	~11,000	1129	41	21	5.9	5.6	−4.5	9.3	4.4
DI^13^C w/ CH_4_	10	−39	5051	34	24	0.9	2.1	−5.0	2.3	NA
	20	−0.6	4905	40	22	3.8	5.5	−5.4	11	NA
	30	21	4829	40	20	5.4	5.4	−5.1	8.1	NA
DI^13^C w/o CH_4_	10	NA	5380	34	24	0.8	1.7	−3.6	3.2	NA
	20	NA	5676	36	25	1.0	1.3	−0.5	4.8	NA
	30	NA	4986	35	25	0.8	0.6	−0.5	3.1	NA
DIC w/ ^13^CH_4_	Control (30)	~12,000	28	4	22	0.0	−0.2	−6.1	−30	0.0
DI^13^C w/o CH_4_	Control (30)	−43	4841	4	22	0.0	−0.2	−6.0	−30	NA
DIC w/ CH_4_	30	−42	−16	41	21	4.5	4.3	−4.7	7.2	0.2
DIC w/ ^13^CH_4_	Blank (30)	~8000	27	34	26	0.1	NA	NA	NA	0.1
DI^13^C w/o CH_4_	Blank (30)	−43	6484	3	26	0.1	NA	NA	NA	NA
DIC w/ CH_4_	Blank (30)	−43	−11	34	26	0.1	NA	NA	NA	NA

*Note:* Listed are *δ* ^13^C values of methane (CH_4_) and dissolved inorganic carbon (DIC), as well as the concentrations of DIC, sulphate (SO_4_^2−^) and hydrogen sulphide (H_2_S). Treatments with “^13^C” stand for a ^13^C labelling of ~10%. Production and consumption rates of SO_4_^2−^, H_2_S, DIC, and CH_4_ are reported in μmol g_dw_^−1^ d^−1^.

Abbreviation: NA, not analysed.

### 2.3 Methane Stable Carbon Isotope Analysis

After completion of the experiment, the headspace methane of all samples was analysed for stable carbon isotope compositions. In brief, 1 mL headspace gas was transferred into a N_2_‐purged 12 mL gas‐tight exetainer vial filled with 1 mL concentrated NaOH (18 M, Merck). Vials were stored upside down at room temperature to minimise gas prior to measurements. The *δ* ^13^C values of methane were determined using gas chromatography (Trace GC ultra) coupled via a GC combustion III interface to a Delta Plus isotope ratio mass spectrometer (IRMS; all ThermoFinnigan). Gas chromatography involved a Carboxen 1006 PLOT fused silica capillary column (30 m × 0.32 mm, Supelco) and helium as carrier gas at a constant flow rate of 3.0 mL min^−1^. 100 μL sample volume was injected with a split ratio of 1:3 at an injector temperature of 200°C. The oven temperature was isothermally set to 40°C for 6 min; the interface, converting CH_4_ into CO_2_, was running at 940°C. *δ* ^13^C values were referenced against a CO_2_ gas (Air Liquide), and the analytical precision was assessed by repeated measurements of a CH_4_ gas standard (Air Products), both with known *δ* ^13^C values. Each sample was analysed in duplicates, which revealed an overall precision of < 0.5‰ for non‐labelled compounds. The carbon isotope composition is expressed relative to the VPDB standard:

(1) $$\delta^{13}C=\left(\frac{{\left(\frac{C^{13}}{C^{12}}\right)}_{\text{sample}}}{{\left(\frac{C^{13}}{C^{12}}\right)}_{\text{Standard}}}-1\;\right)\times 1000$$

### 2.4 DIC Concentration and Stable Carbon Isotope Analysis

To analyse concentration and *δ* ^13^C values of DIC, 550 μL of the incubation medium was filtered (Minisart, 0.22 μm, High Flow PES) into 12‐mL Exetainer vials (Labco), which were filled prior with 100 μL 45% phosphoric acid and purged with helium gas. After equilibration overnight, the released headspace CO_2_ was analysed using a DeltaRay IRIS with URI connect and a Cetac ASX‐7100 Autosampler (Thermo Fisher Scientific). The *δ* ^13^C values were calibrated against an isotopically known CO_2_ reference gas, and the precision (< 0.5‰) was assessed by regular measurements of isotopically known sodium bicarbonate (Sigma‐Aldrich). The resulting CO_2_ volume was converted into a DIC concentration via the gas constant at 25°C and corrected by the instrument's recovery rate of 86%. The carbon isotope composition is expressed as above relative to the VPDB standard (Equation 1).

### 2.5 Sulphide and Sulphate Concentrations

For the analysis of sulphur species, a 1 mL aliquot of filtered incubation medium was fixed with 0.5 mL of 100 mM zinc acetate until measurements. For sulphide measurements, the formed zinc acetate was homogenised in the solution. In subsamples, sulphide was measured using the Cline assay (Cline 1969) and photometric measurement at 670 nm after a 30‐min reaction period in the dark. For sulphate measurements, fixed samples were diluted 1:50, and sulphate concentrations were measured by ion chromatography (930 compact IC flex, Metrohm with Metrosep A Supp 4150/4.0 column).

### 2.6 Lipid Extraction and Derivatization

After incubation, sediment slurries were centrifuged, freeze‐dried (0.5–1 g dry weight, g_dw_), and extracted via a modified Bligh and Dyer protocol (Sturt et al. 2004). Prior to extraction, behenic acid methyl ester and 1‐nonadecanol were added as internal standards. The total lipid extract (TLE) was saponified with 6% potassium hydroxide (KOH, Sigma–Aldrich) in methanol (MeOH, Sigma–Aldrich) reacting for 3 h at 80°C. Subsequently, the product was extracted three times with *n‐*hexane: first under basic conditions to obtain neutral lipids (NL) and then under acidic conditions to obtain fatty acids (FA). Both fractions were dried under a stream of nitrogen and stored at −20°C until further workup. FAs were derivatized with 10% BF_3_ in MeOH (VWR) at 70°C for 1 h forming fatty acid methyl esters (FAMEs) and extracted three times before they were dried under a stream of nitrogen and stored at −20°C before measurements within the next 5 days. NLs, containing hydrocarbons and alcohols, were derivatized using BSTFA (Sigma–Aldrich) in pyridine (anhydrous, 99.8%, Sigma–Aldrich) for 1 h at 70°C forming TMS derivatives of the alcohols. TMS derivatives were measured on the same day to avoid hydrolysis.

### 2.7 Lipid Analysis via GC‐FID, GC–MS and GC‐IRMS

Lipids were analysed by gas chromatography coupled to a flame ionisation detector (GC‐FID; Trace GC Ultra, Thermo Scientific). Their concentrations were determined by comparing peak areas to those of the internal standards. Lipids were qualitatively confirmed by coupled GC‐mass spectrometry (GC–MS; Trace 1310 + ISQ 700, Thermo Scientific) equipped with an electron impact ionization (EI) source set at 70 eV. The single quadrupole was operated in EI full scan mode, acquiring masses from *m*/*z* 50–850. The lipids’ *δ* ^13^C values were determined in duplicate measurements using GC‐IRMS (Trace GC Ultra coupled to a GC‐IsoLink and connected via a ConFlow IV interface to a Delta V Plus IRMS, Thermo Scientific). All GCs were run in splitless injection mode (1 min) at 310°C. They were equipped with the same Restek Rxi‐5 ms column (30 m × 0.25 μm, Restek) and operated with the same temperature program: oven held at 60°C for 1 min, raised to 150°C at a rate of 10°C min^−1^, then to 310°C at a rate of 4°C min^−1^ and the final temperature was held at 310°C for 30 min for NLs and 20 min for FAMEs. Helium was used as carrier gas at a constant flow rate of 1.0 mL min^−1^, except for the GC‐IRMS where 1.2 mL min^−1^ was applied. For GC‐IRMS measurements, the GC‐separated compounds were oxidised to CO_2_ in a combustion reactor operated at 940°C. Calibration of the instrument was performed against an isotopically known CO_2_ reference gas and the precision was assessed by regular measurements of a C_20_–C_40_ *n‐*alkane standard, which has a long‐term variance of ±0.5‰. *δ* ^13^C values of each fraction from non‐13C‐labelled samples showed deviations of < 1‰ between duplicate measurements. *δ* ^13^C values of FAMEs and TMS‐derivatives were corrected for additional carbon introduced during derivatization and are reported in delta notation (*δ* ^13^C) relative to VPDB (Equation 1). Representative GC‐FID runs of the NL and FA fraction are displayed in Figure S1.

### 2.8 Rate Determination of SO_4_^2^ ^−^ Consumption, H_2_S Production, DIC Consumption and CH_4_ Oxidation

SO_4_^2−^, H_2_S and DIC consumption or production rates (Table 1) were calculated by dividing the measured change in concentration in mM (Table 1) by the volume of the liquid (0.1 L) in each incubation bottle, the time of sampling and its corresponding dry weight to yield a rate in μmol g_dw_^−1^ day^−1^.

(2) $$\mathrm{rat}e_{\text{production}/\text{consumption}}=\frac{\Delta \text{concentration}}{V_{\text{medium}}\times g_{\mathrm{dw}}\times \text{time}}$$

Methane oxidation rates were calculated based on the change in *δ* ^13^C values of DIC in the DIC with ^13^CH_4_ experiment, where the initial *δ* ^13^CH_4_ value at day 0 was +10,000‰. During the 30‐day incubation period, there was a detectable increase in the concentration and *δ* ^13^C value of DIC, leading to final values of +9.1 mM and +1140‰, respectively (Figure 3, Table 1). The enrichment in ^13^C was used to calculate the corresponding CH_4_ oxidation rate using Equation (3) (Kellermann et al. 2012):

(3) $$CH_{4}\;\mathrm{ox}.\;\text{rate}=\frac{\;\Delta F^{13}C_{\mathrm{DIC}}\times \mathrm{con}c_{\mathrm{DIC}}}{\text{excess}\;F^{13}C_{CH_{4}}\times \text{time}}$$

In the equation, Δ*F* ^13^C represents the change in the fraction of ^13^C, while the excess $\;F^{13}C_{CH_{4}}$ represents the initial fraction of ^13^C. Methane oxidation rates after 10, 20 and 30 days can be found in Table 1.

### 2.9 Determination of Assimilation Rates and Lipid Turnover Times

The assimilation rate of IC (assim_IC_; Kellermann et al. 2012; Wegener et al. 2012) was calculated by multiplying average lipid concentrations (conc_lipid_) with the increase of ^13^C in the lipids (Δ*F* ^13^C_lipid_) relative to the non‐labelled samples, and divided by the fraction of ^13^C in the incubation medium (*F* ^13^C_medium_) and the incubation time (*t*), as shown in the following equation:

(4) $$\text{assi}m_{\mathrm{IC}}=\mathrm{con}c_{\text{lipid}}\times \frac{\Delta F^{13}C_{\text{lipid}}}{F^{13}C_{\text{medium}}\times t}$$

In this equation, *F* ^13^C refers to the proportion of ^13^C in both the lipid and the medium, calculated from the respective stable carbon isotopic ratios ($F^{13}C=\frac{R^{13C}}{R^{13C}+1}$; 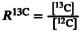). For DI^13^C with CH_4_, we used the measured *δ* ^13^C_DIC_ values, assuming neglectable change (< 1%) in the *δ* ^13^C_DIC_ pool over the course of the incubation. In the experiments of DIC with ^13^CH_4_, we used the average *δ* ^13^C_DIC_ value between day 0 and each day of sampling (10, 20 or 30), because in this treatment *δ* ^13^C_DIC_ increases significantly over time and the discrete measurements at each day of sampling do not reflect the average *δ* ^13^C_DIC_ value.

For calculation of lipid turnover times, we divided the *F* ^13^C_medium_ value by the overall difference in Δ*F* ^13^C_lipid_ and multiplied with the time (*t*).

(5) $$\text{lipid turnover time}=\frac{F^{13}C_{\text{medium}}\times t}{\Delta F^{13}C_{\text{lipid}}}$$

### 2.10 DNA Extraction and Short‐Read Sequencing

DNA was extracted from pellets of 250 mg of sediment samples using the DNeasy PowerSoil Pro kit (Qiagen). Total DNA yield per sample, determined by fluorometric DNA concentration measurement, ranged from 226.5 to 465 μg (4.53 and 9.3 ng μL^−1^; 50 μL per sample). Samples were sequenced at LGC Genomics GmbH (Berlin, Germany). Samples were sequenced as 2 × 150 bp paired‐end reads on an Illumina MiSeq sequencing platform. Between 19,152,757 (Start, day 0) and 20,850,907 (End, day 30) raw reads were obtained (Table S1).

### 2.11 Analysis of Community Composition

Metagenomic raw reads were first quality checked using FastQC v0.11.4 and MultiQC v1.13 (https://github.com/s‐andrews/FastQC; https://github.com/MultiQC/MultiQC) with default settings. Low‐quality reads and adapters were removed from the dataset using BBDuk of the BBtools package v38.98 (https://sourceforge.net/projects/bbmap/; parameters: minlength = 50 qtrim = rtrimq = 20). Microbial community composition based on 16S rRNA gene abundance was calculated with phyloFlash v. 3.3b1 (https://hrgv.github.io/phyloFlash/).

### 2.12 Tetra‐Labelled Oligonucleotide Fluorescence in Situ Hybridization

At the start and the end of the incubation (day 0 and 30) 3 mL of sediment slurries were fixed for 2 h using formaldehyde (2.5%) (Sigma–Aldrich) at room temperature. After fixation, the samples were washed with phosphate‐buffered saline (PBS) at pH 7.2 and stored in a 1:1 mixture of PBS and ethanol at −20°C until further analysis. Tetra‐FISH probes were used to visualise the samples, modifying the previously established protocol (Pernthaler et al. 2002, 2001). A formamide concentration of 50% was used for probe ANME2‐538 with the sequence (5′→3′) GGCTACCACTCGGGCCGC targeting the ANME‐2 cluster with the fluorochromes Alexa Fluor 594 (Treude et al. 2005). Formaldehyde‐fixed sediment samples stored in PBS–ethanol were diluted 10 times with PBS–ethanol and sonicated on ice using a type MS72 probe (Sonopuls HD70; Bandelin, Berlin, Germany; 8 cycles for 30 s with 86% amplitude). 100 μL of the mixture were filtered onto 0.2 μm pore size polycarbonate filters (GTTP, Millipore, Eschborn, Germany) (Ravenschlag et al. 2000). Filters were cut into 10 sections, and each filter section was embedded in 0.1% (w/v) agarose, and dried face down onto a parafilm‐covered plastic plate at 37°C for 30 min. Microbial cell walls were permeabilized with lysozyme solution (10 mg mL^−1^ in 0.05 M EDTA, 0.1 M Tris–HCl (pH 7.5), Fluka). Filter sections were incubated at 37°C for 60 min, followed by treatment with proteinase K solution (0.45 mU mL^−1^ proteinase K in 0.05 M EDTA, 0.1 M Tris–HCl (pH 7.5), 0.5 M NaCl, Fluka) at room temperature for 5 min. Hybridization was carried out for 3 h at 46°C in hybridization chambers. Each filter section received a mixture of 5 μL probe working solution (8.4 pmol μL^−1^) and 45 μL of hybridization buffer (900 mM NaCl, 20 mM Tris–HCl (pH 8.0), 0.01% wt. vol^−1^ sodium dodecyl sulphate (SDS), and 50% (vol/vol) formamide). Following the incubation, the filter sections were washed in 50 mL of pre‐warmed washing buffer (28 mM NaCl, 5 mM EDTA (pH 8.0), 20 mM Tris–HCl (pH 7.5), and 0.01% (wt. vol^−1^) SDS) at 48°C for 15 min. Filter sections were subsequently washed in ultrapure water (MQ; Millipore) and 96% (vol/vol) ethanol, and air‐dried on blotting paper (Whatman, UK). Dried filter sections were mounted with mixtures of CitiFluorAF1 (CitiFluor Ltd., London, UK) and Vectashield (Vector Laboratories, Burlingame, CA, USA) containing 1 μg/mL DAPI (4′,6‐diamidino‐2‐phenylindole; Sigma–Aldrich, Steinheim, Germany).

### 2.13 AI Usage, Data Analysis and Data Visualisation

We employed large language models (LLMs), specifically OpenAI's ChatGPT, for primary code development with respect to data evaluation and/or creation of figures. For the quantification of lipids, the Python package “pyGC_FID_processing” (Zander 2024) was used. To validate compound quantification, we manually integrated a subset of chromatograms to compare them with the automated approach. Data analysis and figure generation were performed in R Studio (RStudio Team 2024) and Tidyverse (Wickham et al. 2019). Figure design was finalized with the vector graphic editor Inkscape V1.4 (86a8ad7, 2024‐10‐11).

## 3 Results

In this study, we used SIP experiments with ^13^CH_4_ and DI^13^C to distinguish between the assimilation of methane or IC into lipid biomass of AOM community members in active cold seep sediments of the Astoria Canyon, Oregon. In addition, we examined the data set for lipid biosynthesis pathways and possible functional roles of lipids.

### 3.1 Analysis and Visualisation of the Natural Microbial Communities

**FIGURE 1:**
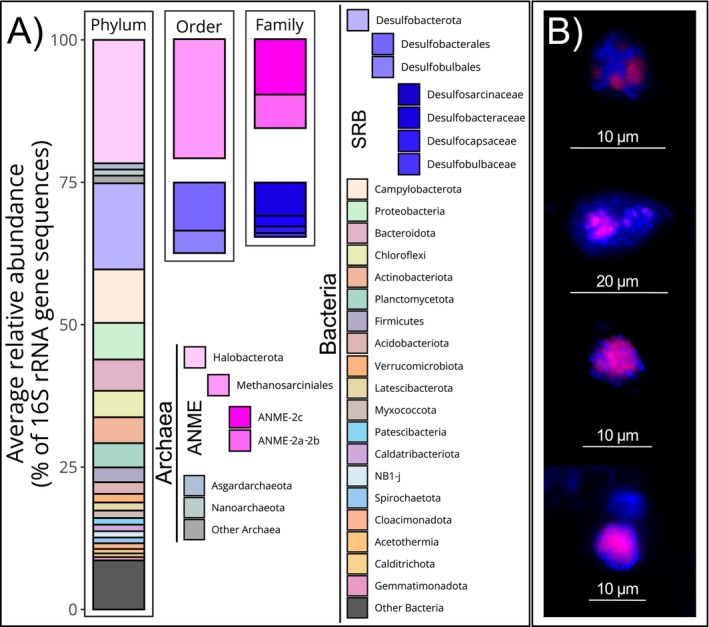
Community analysis and AOM aggregates visualization. (A) Community composition of the cold seep sediment used in the SIP experiment. Average relative abundances of 16S rRNA gene sequences obtained from the metagenome at the start and the end of the SIP experiments are shown (*n* = 2). The panels (from left to right) display annotations at the phylum level of all classified sequences, followed by sequences classified as ANME and SRB at the order and family levels. ANME members are highlighted in magenta, and SRB known to form consortia are highlighted in blue. (B) Fluorescence micrographs (Tetra‐FISH) of the cold seep sediments. Images show cells hybridised with the 538 specific to ANME‐2 (magenta), surrounded by SRB cells only stained with DAPI, together forming the AOM consortia in various shapes and sizes.

To characterise the microbial community of the cold seep sediment, we extracted and sequenced the total DNA metagenome, and taxonomically analysed the recovered 16S rRNA gene sequences (Figure 1A). The relative abundance of the different microbial groups remained largely stable throughout the incubation, with changes of less than 3% on the phylum and order levels, and less than 1.5% on the family level (Table S2). Based on the 16S rRNA gene reads, *Halobacteriota* archaea accounted for 22% of the total community at the phylum level, with *Methanosarcinales* representing 21% at the order level. Within this order, ANME‐2c was the most abundant, accounting for 10%, followed by ANME‐2a/b with 6% (Figure 1A and Table S2). Desulfobacterota constituted 15% at the phylum level, with *Desulfobacterales* and *Desulfobulbales* representing 8% and 6% of the total microbial community. Within these orders, *Desulfosarcinaceae* was the most abundant family, accounting for 6% of the total microbial community (Figure 1A and Table S2). Other major phyla included Campylobacterota (9%), Proteobacteria (6%), Bacteroidota (6%) and Chloroflexi and Actinobacteriota (5% each) (Figure 1A and Table S2).

We performed Tetra‐FISH analysis at the start and end of the incubation to visualise the ANME‐2/SRB consortia (Figures 1B and S2). A specific ANME‐2 probe stained archaeal DNA in magenta, while all DNA was stained with DAPI (blue). The shape and distribution of ANMEs and SRBs in the consortium generally correspond to previously described ANME‐2/SRB consortia observed with FISH microscopy (Knittel and Boetius 2010; Metcalfe et al. 2021). The relatively small size of the observed consortia likely resulted from the harsh ultrasonication step during sample preparation, which fragmented larger aggregates. Nonetheless, the observation of AOM‐SR consortia supports the presence and activity of ANME‐2 and their SRB partners.

### 3.2 Lipid Composition and *δ* ^13^C Values of the Original Cold Seep Sediment

**FIGURE 2:**
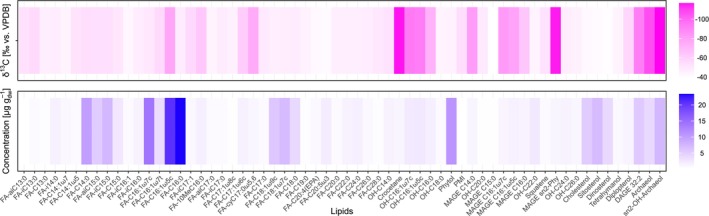
Heat map showing the concentrations (blue; in μg g_gw_^−1^) and *δ* ^13^C values (magenta; in ‰ vs. VPDB) of fatty acids, alcohols and hydrocarbons extracted from the incubated sediment. ai: anteiso, C, carbon chain length, cy: cyclic; DAGE, dialkyl glycerol ether; FA, fatty acid; i: iso; MAGE, monoalkyl glycerol ether; OH, hydroxy.

Lipid concentrations and their *δ* ^13^C values of the original sediment used for the SIP experiment are shown in Figure 2 and Table S3. Crocetane (1.3 μg g_dw_^−1^; *δ* ^13^C −100‰), archaeol (4.0 μg g_dw_^−1^, −91‰), sn2‐OH‐archaeol (7.4 μg g_dw_^−1^, −101‰) and sn2‐phytanyl‐mono‐alkyl‐glycerol ether (sn2‐phy MAGE, 1.2 μg g_dw_^−1^, −99‰) are present in the sediment, which all show strong depletion in ^13^C. The resulting ratio of sn2‐OH‐archaeol to archaeol is 1.9. The following bacterial lipids are also present and have less negative *δ* ^13^C values: MAGE C_16:1ω5c_ (1.3 μg g_dw_^−1^, −66‰), dialkyl glycerol ether (DAGE) C_32:2a_ (5.6 μg g_dw_^−1^, −77‰), and FAs like C_16:1ω5c_ (20.7 μg g_dw_^−1^, −61‰) and cyC_17:0ω5,6_ (1.1 μg g_dw_^−1^, −59‰, Figure 2, Table S3). Other abundant bacterial lipids have more positive *δ* ^13^C values: C_14:0_ (9.9 μg g_dw_^−1^, −33‰), iC_15:0_ (7.3 μg g_dw_^−1^, −38‰), aiC_15:0_ (5.3 μg g_dw_^−1^, −38‰), C_16:0_ (23.4 μg g_dw_^−1^, −33‰), and C_18:1ω7c_ (7.0 μg g_dw_^−1^, −32‰). Moreover, the sediment shows strong marine input of algal biomarker with typical isotopic compositions such as phytol (10.9 μg g_dw_^−1^, −29‰), cholesterol (5.3 μg g_dw_^−1^, −26‰), sitosterol (7.0 μg g_dw_^−1^, −26‰) and dinosterol (4.0 μg g_dw_^−1^, −24‰).

### 3.3 Development of AOM Activity During Incubation

**FIGURE 3:**
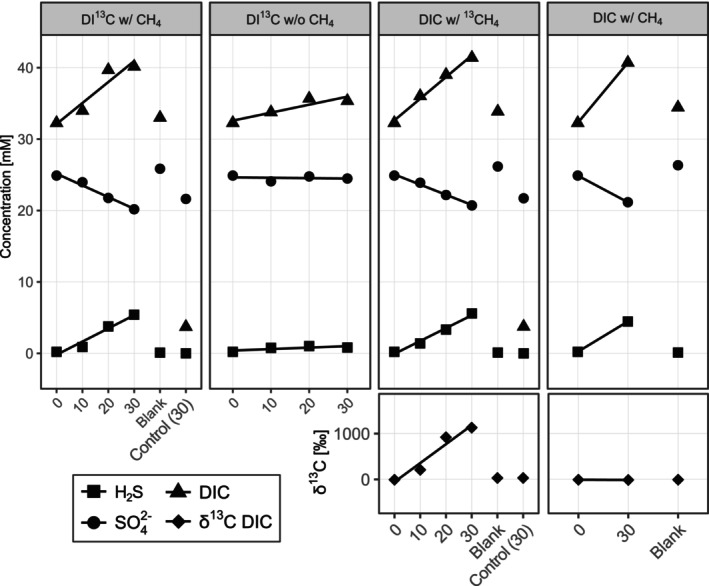
Development of chemical parameters in the four main treatments between 0 and 30 days. The first upper four panels show dissolved inorganic carbon (DIC), sulphate (SO_4_^2−^) and hydrogen sulphide (H_2_S) concentrations in mM; the two lower panels display *δ* ^13^C values of DIC in ‰. All treatments with (w/) methane (CH_4_) show an increase over time, while the treatment without (w/o) CH_4_ does not. The treatment with ^13^C‐labelled CH_4_ shows steady label transfer into the DIC pool.

Changes in geochemical data (*δ* ^13^C values of CH_4_ and DIC, concentrations of DIC, SO_4_^2−^ and H_2_S) during the SIP experiment indicate sulphate‐dependent AOM as the dominant microbial process (Table 1, Figure 3). Because methane was provided in excess (1 bar) we did not measure its concentration. In the ^13^CH_4_‐labelling experiment, the *δ* ^13^C‐DIC values increased by 1140‰ within 30 days. Based on this shift in *δ* ^13^C‐DIC, we calculated a methane oxidation rate of approximately 4.4 μmol g_dw_^−1^ d^−1^. In the same treatments, sulphate decreased by 4.8 ± 0.3 μmol g_dw_^−1^ d^−1^ (mean and SD, *n* = 3) while sulphide increased by 5.1 ± 0.7 μmol g_dw_^−1^ d^−1^ (mean and SD, *n* = 3). The mismatch between sulphate reduction and methane oxidation rates can be explained by additional organoclastic sulphate reduction. In all treatments, the DIC production rate (8.1 ± 1.1 μmol g_dw_^−1^ d^−1^, mean and SD, *n* = 3) was higher than the methane oxidation rate (4.8 ± 0.3 μmol g_dw_^−1^ d^−1^), suggesting dissolution of inorganic carbon. Notably, in the DI^13^C treatments, the *δ* ^13^C–CH_4_ values increased by 60‰. Such label transfer into the CH_4_ pool is known due to the reversibility of the intracellular reactions during AOM (Holler et al. 2011; Wegener et al. 2012).

### 3.4 Lipid‐Specific Labelling With DI^13^C and ^13^CH_4_

**FIGURE 4:**
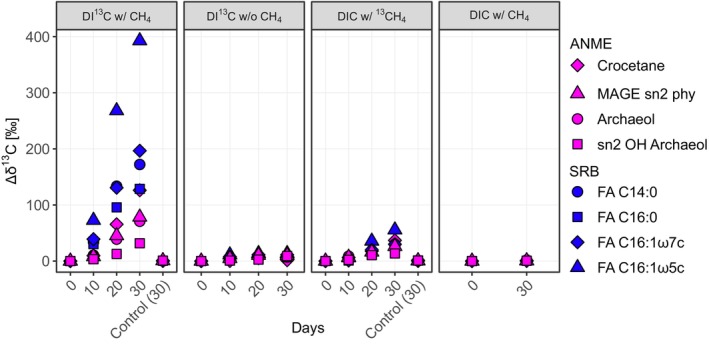
Shifts in *δ* ^13^C values (Δ*δ* ^13^C) of lipid biomarkers in the SIP incubation experiments. Each panel represents one treatment over time, from left to right: DIC with ^13^CH_4_, DI^13^C with CH_4_, DI^13^C without CH_4_ and non‐labelled DIC with CH_4_. Colours represent lipids diagnostic for ANME (magenta) and the most strongly ^13^C‐labelled lipids diagnostic for SRB (blue) grouped by compound classes, that is, FAs, MAGEs and DAGEs. Shapes represent individual lipid biomarkers and are consistent within each microbial group. FA, fatty acid; OH, hydroxy.

Throughout the incubation, concentrations of individual lipids remained largely stable, which is consistent with the slow growth of the AOM consortia. Nevertheless, diagnostic lipid biomarkers of ANME‐2 and SRB showed substantial assimilation of the supplied ^13^C‐labelled carbon sources (Figure 4, Table S4). We observed the most pronounced ^13^C assimilation in the SRB‐specific fatty acids C_16:1ω5c_ and cyC_17:0ω5,6_ with changes in the stable carbon isotopic composition (Δ*δ* ^13^C‐values) of +393‰ and + 258‰, respectively, in the DI^13^C w/ CH_4_ experiment after 30 days. Other fatty acids (C_14:0_, C_16:1ω7c_ and C_16:0_) were also strongly ^13^C‐labelled reaching Δ*δ* values of +172‰, +197‰, and +129‰, respectively. Of the archaeal lipids, Δ*δ* ^13^C‐values were highest for crocetane (+126‰) and MAGE sn2‐phy (+78.4‰). In experiments without methane (DI^13^C w/o CH_4_), the DI^13^C‐label assimilation was minimal: archaeal lipids showed Δ*δ* ^13^C‐values between +0.3‰ and +10.4‰, while bacterial lipids ranged from +6.6‰ to +13.5‰. In experiments with ^13^CH_4_ addition, SRB‐specific lipids reached Δ*δ* ^13^C values of +55.6‰ (C_16:1ω5c_) and +32.8‰ (cyC_17:0ω5,6_) and archaeal lipids reached Δ*δ* ^13^C values of +36.9‰ for crocetane and +27.7‰ for archaeol. Other more general bacterial fatty acids such as *ai*C_15:0_ and *i*C_15:0_ showed Δ*δ* ^13^C values of +21.3‰ and +14.1‰, respectively, in the DI^13^C w/o CH_4_ treatment. These values were only slightly higher in the DI^13^C with CH_4_ experiment (Δ*δ* ^13^C values +37.8‰ and +28.8‰, respectively).

### 3.5 DIC And Methane Assimilation Rates Into AOM Specific Lipids

**FIGURE 5:**
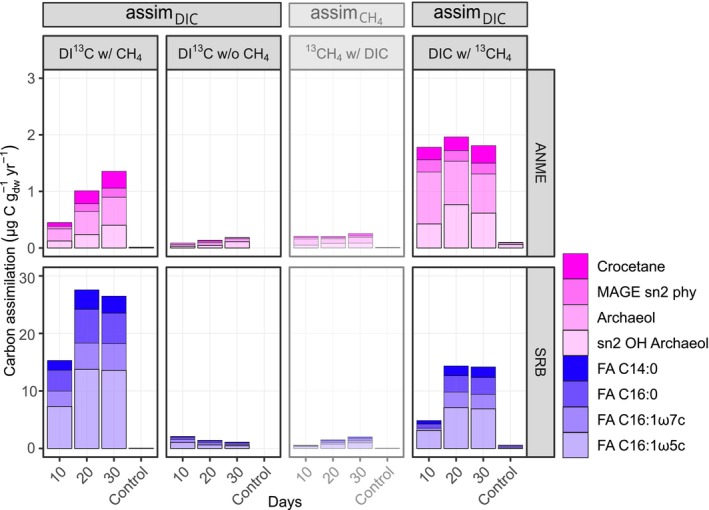
Incorporation of carbon from dissolved inorganic carbon (DIC) and methane into diagnostic lipids of AOM‐associated microorganisms across stable isotope probing (SIP) treatments. Vertical panels represent individual SIP treatments: From left to right (1) DI^13^C with CH_4_, (2) DI^13^C without CH₄. Both treatments show inorganic carbon assimilation (assim_IC_) rates. (3) ^13^CH₄ with DIC. This treatment shows potential methane carbon assimilation (assim_CH4_) with reduced opacity. (4) DIC with ^13^CH_4_. This treatment shows inorganic carbon assimilation (assim_IC_). Horizontal panels separate microbial groups: Anaerobic methanotrophic archaea (ANME, top) and sulphate‐reducing bacteria (SRB, bottom). Bar heights indicate carbon assimilation in μg C g_dw_ year^−1^ into diagnostic lipids after 10, 20, and 30 days. Where available, 30‐day values for killed controls are shown for comparison. Lipid classes include fatty acids (FA), and hydroxy (OH) groups. The shading represents individual compounds.

We calculated assim_IC_ for treatments with labelled DI^13^C and all time points according to Equation (4). In the DI^13^C w/ CH_4_ incubations, SRB lipids showed the highest assim_IC_ values. For example, C_16:1ω5c_ assimilated 13.6 μg C g_dw_^−1^ year^−1^ and C_16:0_ assimilated 5.3 μg C g_dw_^−1^ year^−1^ calculated from values for the 30 days SIP incubation experiment (Figure 5, Table S5). Archaeal lipids also assimilated substantial ^13^C‐label in the same DI^13^C w/ CH_4_ treatment based on the 30days incubation period: archaeol assimilated 0.49 μg C g_dw_^−1^ year^−1^ and sn2‐OH‐archaeol assimilated 0.24 μg C g_dw_^−1^ year^−1^. In the treatment without methane (DI^13^C w/o CH_4_, Figure 5, Table S5), ^13^C‐DIC assimilation into lipids dropped substantially. After 30 days, assim_IC_ in SRB lipids was on average 94% ± 4% (mean and SD, *n* = 4) and in ANME‐2 archaeal lipids 87% ± 11% (mean and SD, *n* = 4) lower compared to the conditions with methane. By comparison, lipids most likely produced by the heterotrophic background community (i.e., *ai*C_15:0_, *i*C_15:0_, and C_18:1ω7c_) showed overall lower assim_IC_ values (0.2–0.4 μg C g_dw_^−1^ year^−1^). Notably, ^13^C‐assimilation into these lipids was less affected by the absence of methane (only ~57% ± 15% (mean and SD, *n* = 3) reduction compared to the presence of methane).

To assess the role of methane as a carbon source, we calculated assim_CH₄_ values exclusively for treatments where CH₄ was ^13^C‐labelled (Figure 5, lesser opacity, Table S6). Such calculations were not considered in any lipid SIP study yet and values for SRB are only hypothetical due to their sole autotrophic carbon fixation lifestyle. These calculations revealed that carbon assimilation from methane would be substantially lower than from DIC, particularly in archaeal lipids. For instance, in the DIC w/^13^CH_4_ treatment, only 0.1 μg gdw^−1^ year^−1^ of archaeol could be attributed to methane as a carbon source, which is similar to the assim_CH₄_ value for archaeol in the killed control of the same treatment after 30 days. To compare assim_IC_ and assim_CH₄_ rates during the treatment DIC w/^13^CH_4_, we used the averaged *δ* ^13^C‐DIC values, which increased to 1129‰ during the 30 days experiment to calculate assim_IC_. Those derived assim_IC_ values were on the same magnitude as those during the DI^13^C w/ CH_4_ treatments (Figure 5, right panel, Table S5). Values for ANME markers crocetane, sn2‐phy MAGE, archaeol and sn2‐OH‐archaeol were between 0.2 and 0.7 μg C g_dw_^−1^ year^−1^ and values for SRB FA C_16:1ω5c_ and C_16:0_ were 2.9 and 6.9 μg C g_dw_^−1^ year^−1^ (Table S5).

The temporal dynamics of calculated individual and summed assim_IC_ values differed on the labelled substrates (DI^13^C vs. ^13^CH_4_) and between the organism groups (ANME vs. SRB lipids, Figure 5, Table S5). In the DIC w/^13^CH_4_ experiment, the assim_IC_ into summed ANME lipids was largely stable at about 1.5 μg gdw^−1^ year^−1^ for all three observation periods. By contrast, in the DI^13^C w/ CH_4_ experiment, the calculated DI^13^C assimilation into ANME lipids was lower for the 10‐day period, and increased to almost 1.5 μg gdw^−1^ year^−1^ for the 30‐day period. SRB‐specific lipids showed similar developments of assim_IC_ values during the course of both experimental types. In the treatment DI^13^C w/ CH_4_, summed bacterial FAs exhibited ^13^C assimilation values of around 15 μg gdw^−1^ year^−1^ for the 10‐day period, which increased to ~30 μg gdw^−1^ year^−1^ at the other time points. The same temporal pattern of the assim_IC_ values was observed for the DIC w/^13^CH_4_ treatment for partner bacteria‐specific lipids, but the assim_IC_ values were only half as high.

### 3.6 Lipid Turnover Times During SIP With DI^13^C

ANME lipid turnover times obtained according to Equation (5) ranged from 3.6 to 14.5 years, while those for the lipids of their SRB partner were shorter, around 1–7 years (Table S7). At the final incubation point, archaeal lipids crocetane, archaeol, and sn2‐Phy‐MAGE had turnover times of 3.6, 5.9 and 6.5 years. In contrast, sn‐2‐OH‐archaeol exhibited a notably longer turnover time of ~13.3 years. Among the SRB lipid markers, the fatty acid C_16:1ω5c_ displayed the shortest turnover time (1.2 years). Other SRB lipid markers showed intermediate turnover times (~1.8–7.8 years), including MAGE C_16:1ω5c_, DAGE C_32:2_, and the more ubiquitous fatty acids C_14:0_ and C_16:0_. These longer turnover times might indicate either a higher fossil lipid contribution or, particularly in the case of C_14:0_ and C_16:0_, reduced biomarker specificity since various other bacterial groups also synthesize these fatty acids.

## 4 Discussion

Metagenomic and microscopy analyses provide evidence that the microbiome of the methane‐rich cold seep sediment in Astoria Canyon is dominated by archaea of the ANME‐2 clade and their syntrophic SRB partners (Figure 1). Together, ANME‐2c and ANME‐2a/b comprised on average 15.6% of the community at the family level, while *Desulfobacterota* level—particularly *Desulfosarcinaceae*—accounted for 15.1% at the phylum level (Table S2). The co‐existence of ANME‐2a/b and ANME‐2c with SRB at an approximate 1:1 level is often observed at methane seep locations with high AOM activity like the Eel River Basin (Pernthaler et al. 2008), Hydrate Ridge (Boetius et al. 2000; Treude et al. 2003), and the Black Sea (Michaelis et al. 2002). The shape and spatial arrangement of AOM consortia in those environments closely resemble the Tetra‐FISH micrographs of the Astoria Canyon sediment community presented here (Figure 1).

The presence of strongly ^13^C‐depleted archaeol, sn2‐OH‐archaeol, and crocetane as diagnostic lipid biomarkers further supports the dominance of ANME‐2 (e.g., Blumenberg et al. 2004; Elvert et al. 2005; Niemann and Elvert 2008). The sn2‐OH‐Ar to archaeol ratio of 1.9 in our samples is also consistent with ANME‐2 and distinct from ANME‐1. In addition, SRB‐specific lipids are abundant. These include C_16:1ω5c_ and cyC_17:0ω5,6_ and their respective MAGE, alcohol and DAGE derivates of C_16:1ω5c_. They also exhibit low *δ* ^13^C‐values, although not as strongly ^13^C‐depleted as the lipids produced by ANME‐2. This pattern is consistent with previous findings in methane‐rich seep sediments (e.g., Hinrichs et al. 2000; Elvert et al. 2003, 2005; Blumenberg et al. 2005), in which authors also found a clear isotopic difference between more ^13^C‐depleted ANME lipids and slightly less ^13^C‐depleted SRB lipids. The heterotrophic background community, including members of *Campylobacterota* and *Bacteroidota* closely resembles those found in other seep ecosystems (Pop Ristova et al. 2015; Zhu et al. 2022). These organisms likely produce C_18:1ω9c_, *ai*C_15:0_ and *i*C_15:0_ which have moderately low *δ* ^13^C‐values between −30‰ and −38‰. These lipids show methane‐independent DI^13^C assimilation (Table S5), suggesting that the heterotrophic community grows on a mix of degraded sedimentary organic matter and necromass of the AOM core community (Zhu et al. 2022).

We found that assim_IC_ was about one magnitude greater than ^13^CH_4_ assimilation (assim_CH4_) into diagnostic lipids of both ANME and SRB (Figure 5, Tables S5 and S6). Minor assim_CH4_ values likely reflect assimilation of methane‐derived DIC, because the DI^13^C assimilation calculated for the DIC w/^13^CH_4_ treatment was comparable to those in the DI^13^C w/ CH_4_ treatment (Figure 5, Table S5). These results underline previous SIP studies using only ^13^C‐labelled methane (Blumenberg et al. 2005; Jagersma et al. 2009), and refine the conclusions of Wegener et al. (2008), whose long‐term incubations may have masked distinct assimilation pathways due to equilibration between methane and DIC/CO_2_ pools. The ANME‐2/SRB consortia therefore mainly use DIC as a carbon substrate for their lipid biomass, which is in line with findings in Bertram et al. (2013) and Wegener, Krukenberg, et al. (2016).

The IC assimilation into lipids attributed to SRB after 30 days was about eight times higher than that into ANME‐2 lipids (Figure 5, Table S5; summed assim_IC_ values for archaeal versus bacterial lipids (*n* = 4)). Similar ratios between SRB and ANME‐2 lipid production have been observed before (Bertram 2012; Blumenberg et al. 2005; Wegener et al. 2008). Such differences in assimilation result in shorter turnover times for SRB lipids (1–7.8 years) compared to ANME‐2 derived lipids (3.6–13.3 years). This is counterintuitive, as SRB and ANME typically occur in a ratio of approximately 1:1 in AOM consortia. Moreover, cell‐specific ammonium uptake rates suggest similar growth rates for ANME and SRB (Scheller et al. 2016). A likely explanation for the underestimated ANME‐2 lipid production is that ANME continuously dilute the ^13^C‐DIC signal through the intercellular production of methane‐derived unlabelled CO_2_. In other words, during the DI^13^C labelling experiment, ANME cells experience the externally measured average DI^13^C value, but are internally exposed to a ^13^C‐depleted CO_2_ pool due to continuous AOM. To quantify this effect, we recalculated assim_IC_ using the *δ* ^13^C‐DIC values from the ^13^CH_4_ experiment, where internally methane‐derived ^13^C‐labelled CO_2_ dominates. Using this approach, archaeal lipid production rates increase by approximately 1.8 times after 30 days (Figure 5, Table S5, summed assim_IC_ values for archaeal lipids (*n* = 4)), suggesting that the initial estimates likely underestimated their true biosynthetic activity. Nonetheless, even under these assumptions, the calculated production of all archaeal lipids remained far lower than that of bacterial fatty acids (Figure 5).

An alternative explanation for the underestimation of archaeal lipid production could be the assimilation of additional but yet undetected carbon sources by ANME‐2. Previous studies investigated whether the addition of small organic compounds such as acetate affects AOM rates and consortia growth, yet no positive effects of these organic compounds were found (Nauhaus et al. 2002; Wegener, Kellermann, and Elvert 2016). Moreover, the low *δ* ^13^C values of ANME‐lipids and biomass exclude the assimilation of background organic matter with *δ* ^13^C values typically ≥ −30‰. When ANME assimilate organic carbon sources, these may rather originate from their syntrophic partner bacteria, possibly in the form of metabolic intermediates. Such potential metabolic symbiosis has recently been identified for methanol as a catabolic intermediate between bacteria and methanogens (Huang et al. 2025). Indications in this direction also come from Bertram et al. (2013), who observed ^13^C‐label incorporation from methanol into archaeal lipids in an ANME‐2‐dominated microbial mat, either with or without methane, suggesting that methanol may be a suitable substrate for such cross‐feeding. Future studies should test the interspecies transfer of methanol or other small organic compounds as potential exchanged carbon sources in AOM consortia. Identifying such intermediates could significantly refine our understanding of carbon cross‐feeding and the metabolic interdependence within AOM consortia.

The temporal development of Δ*δ* ^13^C values and carbon assimilation rates in the individual SIP experiments provides insight into lipid biosynthesis pathways and functional roles of lipid compounds. In the SIP experiment with DI^13^C and methane (Tables S4 and S5), the *δ* ^13^C values of the ANME‐2 lipids MAGE sn2‐Phy, archaeol and crocetane, were already much more positive after 10 days, and continued to increase throughout the experiment, indicating immediate and prolonged biosynthesis. MAGE sn2‐Phy is probably formed as a by‐product of the geranylgeranyl reductase–catalysed hydrogenation of geranylgeranylglyceryl phosphate (GGGP). This reaction occurs before the formation of digeranylgeranylglyceryl phosphate (DGGGP) in the archaeol biosynthesis pathway (Caforio and Driessen 2017; Jain 2014; Villanueva et al. 2014). In contrast, sn2‐OH‐archaeol exhibited both, delayed and substantially weaker ^13^C‐label incorporation, which indicates much longer turnover times. This is likely caused by the later hydration of the 2′,3′ double bond in the sn‐2 chain of DGGGP (Mori et al. 2015). The differences in ^13^C‐label incorporation in our experiments are in line with intermediates and products of the lipid biosynthetic route and hence explain the wide range of lipid turnover times in ANME‐2. This also resembles the pattern seen in ANME‐1, where the less abundant and highly ^13^C‐labelled intermediate phosphatidylglycerol‐archaeol is rapidly produced and progressively converted to dominant but less ^13^C‐labelled GDGTs (Kellermann et al. 2016). In summary, when interpreting the ^13^C‐labelling of lipids, both lipid production and transformation pathways must be considered, especially in slow‐growing microorganisms such as AOM consortia.

The immediate and continuous ^13^C‐labelling of crocetane is intriguing and points to an important role in ANME‐2 cell membranes. To date, no definite biosynthetic pathway has been established for the isoprenoidal hydrocarbon formation in archaea. However, there is growing evidence for squalene biosynthesis via the condensation of two farnesyl pyrophosphate units (Rao and Driessen 2024; Salvador‐Castell et al. 2019), possibly mediated by squalene synthase acquired by lateral gene transfer (Santana‐Molina et al. 2020), and followed by hydrogenation to form squalane. By analogy, crocetane may be synthesized through the fusion of two geranyl pyrophosphate units to form fully unsaturated crocetene, which is subsequently reduced (Elvert 1999). This hypothesis is supported by the detection of intermediate hydrocarbons with one or two double bonds in both natural AOM environments and enrichment cultures (Blumenberg et al. 2004; Elvert et al. 2000; Nauhaus et al. 2007; Pancost et al. 2000). The physiological function of isoprenoidal hydrocarbons in archaea remains under debate. However, current evidence suggests that lipid bilayer‐forming archaea insert these non‐polar hydrocarbons into the midplane to regulate membrane properties, such as enhancing fluidity and reducing proton permeability (cf. LoRicco et al. 2023; Salvador‐Castell, Brooks, et al. 2021; Salvador‐Castell, Golub, et al. 2021; Salvador‐Castell et al. 2020), similar to the roles of steroids in eukaryotes and hopanoids in bacteria. Apolar hydrocarbons may therefore represent a strategy by which bilayer‐forming archaea could adapt to extreme environmental and energy constraints without synthesizing bipolar, membrane‐spanning, monolayer‐forming lipids.

Reports on the biosynthesis of ether lipids, that is, MAGEs and DAGEs, in SRBs remain scarce. However, using a culture of a mesophilic alkene‐degrading sulphate reducer, Grossi et al. (2015) provided compelling evidence for a systematic link between the chain length and the methyl‐branching pattern of fatty acids, MAGEs and DAGEs. They proposed an unprecedented biosynthetic pathway via primary fatty acids leading to structurally similar MAGEs and DAGEs in anaerobic bacteria. This appears to extend to SRBs involved in AOM, where fatty acid and MAGE patterns—as well as the side chains of corresponding DAGEs—are systematically congruent (e.g., Elvert et al. 2005; Hinrichs et al. 2000). In our current SIP experiments, the biosynthetic trend is evident through the highest ^13^C‐assimilation observed in the fatty acid C_16:1ω5c_, which appears to propagate along the biosynthetic pathway via MAGE finally yielding DAGE C_32:2_ as a probable end product (Tables S4 and S5). DAGE C_32:2_ likely consists of two alcohol chains with a C_16:1ω5c_ structure (Elvert et al. 2005), although an incorporation of C_16:1ω7c_ cannot be ruled out, given its abundance and similar ^13^C‐depletion in the original sediment (Figure 2 and Table S3). To date, enzymes involved in the formation of ether lipids in anaerobic bacteria have rarely been characterised. An exception is key enzymes in *Thermoanaerobacter* (phylum Firmicutes), which can synthesise membrane‐spanning lipids by fusion of two *i*C_15:0_ fatty acids and subsequent reduction to form non‐isoprenoidal ether lipids (Sahonero‐Canavesi et al. 2022). Similar to archaea, the functional role of ether lipid production in bacteria likely relates to enhanced membrane stability. In the context of AOM, this stabilisation, especially in cooperation with ANMEs, may contribute to greater energy efficiency.

## 5 Conclusion

We followed the assimilation of ^13^C‐label into individual lipids of AOM consortia dominated by ANME‐2 and SRB of the order *Desulfobacterales* and *Desulfobulbales* during SIP incubations with DI^13^C or ^13^CH_4_ and calculated lipid‐specific assimilation rates and turnover times to identify their carbon source. The data showed that IC is incorporated far more efficiently into lipid biomass than methane carbon in both ANME‐2 and their SRB partners. Furthermore, SRB lipids assimilated eight times more carbon compared to archaeal lipids. Examination of the temporal pattern of ^13^C‐label incorporation in experiments with DI^13^C suggests that the low carbon assimilation in archaeal lipids may be due to additional fixation of internally generated CO_2_ from ^13^C‐label‐free methane before it can be exchanged with the environment. Because this effect still cannot fully account for the observed differences in archaeal versus bacterial lipid production, we propose that ANME‐2 rely on another carbon source, for example, small organic compounds provided by their SRB partners. Finally, the stepwise ^13^C‐enrichment of lipids of AOM consortia tracks biosynthetic pathways forming diether lipids and specifically suggests crocetane to be an important lipid hydrocarbon that affects membrane function.

## Author Contributions

G.W., M.E., and L.S. conceived and designed the study. L.S. and G.W. conducted the stable isotope probing experiments. L.S. performed lipid extraction, compound‐specific isotope analyses, and data interpretation. Y.W. carried out DNA extraction, microbial community analysis, and tetra‐FISH. Y.Z. contributed to analytical discussions and interpretation. L.S., G.W., and M.E. wrote the manuscript. All authors reviewed and approved the final version of the manuscript.

## Funding

This work was supported by Deutsche Forschungsgemeinschaft, EXC 2077—390741603 and National Science Foundation, 2049517.

## Ethics Statement

All necessary permissions for field sampling were obtained from the relevant national and institutional authorities. No experiments were conducted on humans or vertebrate animals. Research complied with all applicable international, national, and institutional guidelines for marine scientific research, including those related to the Convention on Biological Diversity and the Nagoya Protocol.

## Conflicts of Interest

The authors declare no conflicts of interest.

## Supporting information


**Table S1:** Summary statistics for raw and assembled metagenomes of the unlabelled samples at the start (0 days) and the end (30 days) of the incubation.
**Table S2:** Summary of 16S rRNA gene sequences recruited from the metagenome at the phylum, order and family levels, used to generate the plot in Figure 2A. The data were obtained from the unlabelled treatment at the start (0 days) and the end (30 days) of the incubation. All values are given as relative abundances (%). AVG indicates the average of the start and the end relative abundance values (*n* = 2).
**Table S3:** Concentrations and *δ* ^13^C values of fatty acids, alcohols and hydrocarbons from the original cold seep sediment used for incubation. Quantification was based on internal standards of known concentration added prior to extraction. DAGE, dialkyl glycerol ether; MAGE, monoalkyl glycerol ether; OH, hydroxy.
**Table S4:** Shift of *δ* ^13^C values (Δ*δ* ^13^C, ‰) of lipid biomarkers characteristic for the AOM performing microorganisms in all treatments with the different ^13^C‐labelled and non‐labelled carbon sources. DAGE, dialkyl glycerol ether; FA, fatty acid; MAGE, monoalkyl glycerol ether; OH, hydroxy.
**Table S5:** Carbon assimilation rates in μg C g_dw_^−1^ year^−1^ of characteristic lipid biomarkers calculated with DIC as ^13^C source for all treatments. DAGE, dialkyl glycerol ether; FA, fatty acid; MAGE, monoalkyl glycerol ether; OH, hydroxy.
**Table S6:** Carbon assimilation rates in μg C g_dw_^−1^ year^−1^ of lipid biomarkers characteristic for AOM communities calculated with CH_4_ as ^13^C source. Values for bacterial lipids are hypothetical as SRB are autotrophs. DAGE, dialkyl glycerol ether; FA, fatty acid; MAGE, monoalkyl glycerol ether; OH, hydroxy.
**Table S7:** Calculated lipid turnover times in years of characteristic lipid biomarkers calculated with DIC as ^13^C source for all treatments. DAGE, dialkyl glycerol ether; FA, fatty acid; MAGE, monoalkyl glycerol ether; OH, hydroxy.
**Figure S1:** Partial gas chromatograms of (A) the fatty acid (FA) fraction (analysed as fatty acid methyl esters) and (B) the neutral lipid (NL) fraction, containing alcohols as trimethylsilyl derivatives and hydrocarbons, obtained from the original sediment material used for the SIP experiments.
**Figure S2:** Selection of fluorescence micrographs of the natural AOM enrichment at the start (0 days) and end (30 days) of the incubations. Each image depicts DAPI‐‐stained DNA (all cells, blue) and ANME‐2 specific probe‐stained archaeal cells (red).

## Data Availability

All raw reads generated in this study have been deposited at ENA—BioProject [PRJNA1338113]. Additional data (isotopic values, lipid concentrations and further calculations) are available in the supplemenatry material (Table S1–S7 and Figure S1–S2).

## References

[emi70213-bib-0001] Baumberger, T. , R. W. Embley , S. G. Merle , M. D. Lilley , N. A. Raineault , and J. E. Lupton . 2018. “Mantle‐Derived Helium and Multiple Methane Sources in Gas Bubbles of Cold Seeps Along the Cascadia Continental Margin.” Geochemistry, Geophysics, Geosystems 19: 4476–4486. 10.1029/2018GC007859.

[emi70213-bib-0003] Bertram, S. 2012. Tracing carbon fluxes within two distinct microbial communities in anaerobically methane oxidising mats by stable isotope probing (Dissertation). University of Hamburg.

[emi70213-bib-0002] Bertram, S. , M. Blumenberg , W. Michaelis , M. Siegert , M. Krüger , and R. Seifert . 2013. “Methanogenic Capabilities of ANME‐Archaea Deduced From ^13^C‐Labelling Approaches.” Environmental Microbiology 15: 2384–2393. 10.1111/1462-2920.12112. 23530864

[emi70213-bib-0005] Blumenberg, M. , R. Seifert , J. Reitner , T. Pape , and W. Michaelis . 2004. “Membrane Lipid Patterns Typify Distinct Anaerobic Methanotrophic Consortia.” Proceedings of the National Academy of Sciences of the United States of America 101: 11111–11116. 10.1073/pnas.0401188101. 15258285 PMC503748

[emi70213-bib-0004] Blumenberg, M. , R. Seifert , K. Nauhaus , T. Pape , and W. Michaelis . 2005. “In Vitro Study of Lipid Biosynthesis in an Anaerobically Methane‐Oxidizing Microbial Mat.” Applied and Environmental Microbiology 71: 4345–4351. 10.1128/AEM.71.8.4345-4351.2005. 16085823 PMC1183335

[emi70213-bib-0006] Boetius, A. , K. Ravenschlag , C. J. Schubert , et al. 2000. “A Marine Microbial Consortium Apparently Mediating Anaerobic Oxidation of Methane.” Nature 407: 623–626. 10.1038/35036572. 11034209

[emi70213-bib-0007] Boschker, H. t. s. , and J. j. Middelburg . 2002. “Stable Isotopes and Biomarkers in Microbial Ecology.” FEMS Microbiology Ecology 40: 85–95. 10.1111/j.1574-6941.2002.tb00940.x. 19709215

[emi70213-bib-0008] Caforio, A. , and A. J. M. Driessen . 2017. “Archaeal Phospholipids: Structural Properties and Biosynthesis.” Biochimica et Biophysica Acta 1862: 1325–1339. 10.1016/j.bbalip.2016.12.006. 28007654

[emi70213-bib-0009] Chadwick, G. L. , C. T. Skennerton , R. Laso‐Pérez , et al. 2021. “Comparative genomics reveals electron transfer and syntrophic mechanisms differentiating methanotrophic and methanogenic archaea.” PLoS Biology 20: e3001508. 10.1101/2021.09.25.461819. PMC9012536 34986141

[emi70213-bib-0010] Chadwick, G. L. , C. T. Skennerton , R. Laso‐Pérez , et al. 2022. “Comparative Genomics Reveals Electron Transfer and Syntrophic Mechanisms Differentiating Methanotrophic and Methanogenic Archaea.” PLoS Biology 20: e3001508. 10.1371/journal.pbio.3001508. 34986141 PMC9012536

[emi70213-bib-0011] Claypool, G. E. , and I. R. Kaplan . 1974. “The Origin and Distribution of Methane in Marine Sediments.” In Natural Gases in Marine Sediments, edited by I. R. Kaplan , 99–139. Springer US. 10.1007/978-1-4684-2757-8_8.

[emi70213-bib-0092] Cline, J. D. 1969. “Spectrophotometric Determination of Hydrogen Sulfide in Natural Waters 1.” Limnology and Oceanography 14, no. 3: 454–458. 10.4319/lo.1969.14.3.0454.

[emi70213-bib-0012] Cord‐Ruwisch, R. 1985. “A Quick Method for the Determination of Dissolved and Precipitated Sulfides in Cultures of Sulfate‐Reducing Bacteria.” Journal of Microbiological Methods 4: 33–36. 10.1016/0167-7012(85)90005-3.

[emi70213-bib-0015] Elvert, M. 1999. Microbial Biomarkers in Anaerobic Marine Environments Dominated by Methane (PhD Thesis). Universität Kiel.

[emi70213-bib-0013] Elvert, M. , A. Boetius , K. Knittel , and B. Jørgensen . 2003. “Characterization of Specific Membrane Fatty Acids as Chemotaxonomic Markers for Sulfate‐Reducing Bacteria Involved in Anaerobic Oxidation of Methane.” Geomicrobiology Journal 20: 403–419. 10.1080/01490450303894.

[emi70213-bib-0014] Elvert, M. , E. C. Hopmans , T. Treude , A. Boetius , and E. Suess . 2005. “Spatial Variations of Methanotrophic Consortia at Cold Methane Seeps: Implications From a High‐Resolution Molecular and Isotopic Approach.” Geobiology 3: 195–209. 10.1111/j.1472-4669.2005.00051.x.

[emi70213-bib-0016] Elvert, M. , E. Suess , J. Greinert , and M. J. Whiticar . 2000. “Archaea Mediating Anaerobic Methane Oxidation in Deep‐Sea Sediments at Cold Seeps of the Eastern Aleutian Subduction Zone.” Organic Geochemistry 31: 1175–1187. 10.1016/S0146-6380(00)00111-X.

[emi70213-bib-0017] Goericke, R. , and B. Fry . 1994. “Variations of Marine Plankton δ^13^ C With Latitude, Temperature, and Dissolved CO_2_ in the World Ocean.” Global Biogeochemical Cycles 8: 85–90. 10.1029/93GB03272.

[emi70213-bib-0018] Grossi, V. , D. Mollex , A. Vinçon‐Laugier , F. Hakil , M. Pacton , and C. Cravo‐Laureau . 2015. “Mono‐ and Dialkyl Glycerol Ether Lipids in Anaerobic Bacteria: Biosynthetic Insights From the Mesophilic Sulfate Reducer *Desulfatibacillum alkenivorans* PF2803^T^ .” Applied and Environmental Microbiology 81: 3157–3168. 10.1128/AEM.03794-14. 25724965 PMC4393425

[emi70213-bib-0020] Hallam, S. J. , N. Putnam , C. M. Preston , et al. 2004. “Reverse Methanogenesis: Testing the Hypothesis With Environmental Genomics.” Science 305: 1457–1462. 10.1126/science.1100025. 15353801

[emi70213-bib-0021] Haroon, M. F. , S. Hu , Y. Shi , et al. 2013. “Anaerobic Oxidation of Methane Coupled to Nitrate Reduction in a Novel Archaeal Lineage.” Nature 500: 567–570. 10.1038/nature12375. 23892779

[emi70213-bib-0022] Henderson, L. C. , F. Wittmers , C. A. Carlson , A. Z. Worden , and H. G. Close . 2024. “Variable Carbon Isotope Fractionation of Photosynthetic Communities Over Depth in an Open‐Ocean Euphotic Zone.” Proceedings of the National Academy of Sciences of the United States of America 121: e2304613121. 10.1073/pnas.2304613121. 38408243 PMC10927520

[emi70213-bib-0023] Hinrichs, K.‐U. , and A. Boetius . 2002. “The Anaerobic Oxidation of Methane: New Insights in Microbial Ecology and Biogeochemistry.” In Ocean Margin Systems, edited by G. Wefer , D. Billett , D. Hebbeln , B. B. Jørgensen , M. Schlüter , and T. C. E. Van Weering , 457–477. Springer Berlin Heidelberg. 10.1007/978-3-662-05127-6_28.

[emi70213-bib-0024] Hinrichs, K.‐U. , R. E. Summons , V. Orphan , S. P. Sylva , and J. M. Hayes . 2000. “Molecular and Isotopic Analysis of Anaerobic Methane‐Oxidizing Communities in Marine Sediments.” Organic Geochemistry 31: 1685–1701. 10.1016/S0146-6380(00)00106-6.

[emi70213-bib-0025] Holler, T. , G. Wegener , H. Niemann , et al. 2011. “Carbon and Sulfur Back Flux During Anaerobic Microbial Oxidation of Methane and Coupled Sulfate Reduction.” Proceedings of the National Academy of Sciences 108: E1484–E1490. 10.1073/pnas.1106032108. PMC3248532 22160711

[emi70213-bib-0026] Huang, Y. , K. Igarashi , L. Liu , et al. 2025. “Methanol Transfer Supports Metabolic Syntrophy Between Bacteria and Archaea.” Nature 639: 190–195. 10.1038/s41586-024-08491-w. 39880954

[emi70213-bib-0027] Hügler, M. , and S. M. Sievert . 2011. “Beyond the Calvin Cycle: Autotrophic Carbon Fixation in the Ocean.” Annual Review of Marine Science 3: 261–289. 10.1146/annurev-marine-120709-142712. 21329206

[emi70213-bib-0028] Jagersma, G. C. , R. J. W. Meulepas , I. Heikamp‐de Jong , et al. 2009. “Microbial Diversity and Community Structure of a Highly Active Anaerobic Methane‐Oxidizing Sulfate‐Reducing Enrichment.” Environmental Microbiology 11: 3223–3232. 10.1111/j.1462-2920.2009.02036.x. 19703218

[emi70213-bib-0029] Jain, S. 2014. “Biosynthesis of Archaeal Membrane Ether Lipids.” Frontiers in Microbiology 5: 641. 10.3389/fmicb.2014.00641. 25505460 PMC4244643

[emi70213-bib-0030] Kellermann, M. Y. , G. Wegener , M. Elvert , et al. 2012. “Autotrophy as a Predominant Mode of Carbon Fixation in Anaerobic Methane‐Oxidizing Microbial Communities.” Proceedings of the National Academy of Sciences 109: 19321–19326. 10.1073/pnas.1208795109. PMC3511159 23129626

[emi70213-bib-0031] Kellermann, M. Y. , M. Y. Yoshinaga , G. Wegener , V. Krukenberg , and K.‐U. Hinrichs . 2016. “Tracing the Production and Fate of Individual Archaeal Intact Polar Lipids Using Stable Isotope Probing.” Organic Geochemistry 95: 13–20. 10.1016/j.orggeochem.2016.02.004.

[emi70213-bib-0033] Knittel, K. , and A. Boetius . 2009. “Anaerobic Oxidation of Methane: Progress With an Unknown Process.” Annual Review of Microbiology 63: 311–334. 10.1146/annurev.micro.61.080706.093130. 19575572

[emi70213-bib-0032] Knittel, K. , and A. Boetius . 2010. “Anaerobic Methane Oxidizers.” In Handbook of Hydrocarbon and Lipid Microbiology, edited by K. N. Timmis , 2023–2032. Springer. 10.1007/978-3-540-77587-4_147.

[emi70213-bib-0034] Knittel, K. , G. Wegener , and A. Boetius . 2019. “Anaerobic Methane Oxidizers.” In Microbial Communities Utilizing Hydrocarbons and Lipids: Members, Metagenomics and Ecophysiology, edited by T. J. McGenity , 113–132. Springer International Publishing. 10.1007/978-3-030-14785-3_7.

[emi70213-bib-0035] Kurth, J. M. , N. T. Smit , S. Berger , S. Schouten , M. S. M. Jetten , and C. U. Welte . 2019. “Anaerobic Methanotrophic Archaea of the ANME‐2d Clade Feature Lipid Composition That Differs From Other ANME Archaea.” FEMS Microbiology Ecology 95: fiz082. 10.1093/femsec/fiz082. 31150548 PMC6581649

[emi70213-bib-0036] Lalk, E. , J. Pohlman , L. L. Lapham , et al. 2025. Porewater dissolved organic carbon and associated geochemical data for methane seeps in the Cascadia Margin: Astoria Canyon, Barkley Canyon, Hydrate Ridge, and Bullseye Vent 10.26008/1912/BCO‐DMO.959765.1.

[emi70213-bib-0037] Larowe, D. E. , A. W. Dale , and P. Regnier . 2008. “A Thermodynamic Analysis of the Anaerobic Oxidation of Methane in Marine Sediments.” Geobiology 6: 436–449. 10.1111/j.1472-4669.2008.00170.x. 18699783

[emi70213-bib-0038] Laso‐Pérez, R. , V. Krukenberg , F. Musat , and G. Wegener . 2018. “Establishing Anaerobic Hydrocarbon‐Degrading Enrichment Cultures of Microorganisms Under Strictly Anoxic Conditions.” Nature Protocols 13: 1310–1330. 10.1038/nprot.2018.030. 29773905

[emi70213-bib-0039] Lazar, C. S. , R. J. Parkes , B. A. Cragg , S. L'Haridon , and L. Toffin . 2011. “Methanogenic Diversity and Activity in Hypersaline Sediments of the Centre of the Napoli Mud Volcano, Eastern Mediterranean Sea.” Environmental Microbiology 13: 2078–2091. 10.1111/j.1462-2920.2011.02425.x. 21382146

[emi70213-bib-0041] LoRicco, J. G. , I. Hoffmann , A. Caliò , and J. Peters . 2023. “The Membrane Regulator Squalane Increases Membrane Rigidity Under High Hydrostatic Pressure in Archaeal Membrane Mimics.” Soft Matter 19: 6280–6286. 10.1039/D3SM00352C. 37553974

[emi70213-bib-0042] McGlynn, S. E. , G. L. Chadwick , C. P. Kempes , and V. J. Orphan . 2015. “Single Cell Activity Reveals Direct Electron Transfer in Methanotrophic Consortia.” Nature 526: 531–535. 10.1038/nature15512. 26375009

[emi70213-bib-0043] Merle, S. G. , R. W. Embley , H. P. Johnson , et al. 2021. “Distribution of Methane Plumes on Cascadia Margin and Implications for the Landward Limit of Methane Hydrate Stability.” Frontiers in Earth Science 9: 531714.

[emi70213-bib-0044] Metcalfe, K. S. , R. Murali , S. W. Mullin , S. A. Connon , and V. J. Orphan . 2021. “Experimentally‐Validated Correlation Analysis Reveals New Anaerobic Methane Oxidation Partnerships With Consortium‐Level Heterogeneity in Diazotrophy.” ISME Journal 15: 377–396. 10.1038/s41396-020-00757-1. 33060828 PMC8027057

[emi70213-bib-0045] Meyerdierks, A. , M. Kube , I. Kostadinov , et al. 2010. “Metagenome and mRNA Expression Analyses of Anaerobic Methanotrophic Archaea of the ANME‐1 Group.” Environmental Microbiology 12: 422–439. 10.1111/j.1462-2920.2009.02083.x. 19878267

[emi70213-bib-0046] Michaelis, W. , R. Seifert , K. Nauhaus , et al. 2002. “Microbial Reefs in the Black Sea Fueled by Anaerobic Oxidation of Methane.” Science 297: 1013–1015. 10.1126/science.1072502. 12169733

[emi70213-bib-0047] Mori, T. , K. Isobe , T. Ogawa , T. Yoshimura , and H. Hemmi . 2015. “A Phytoene Desaturase Homolog Gene From the Methanogenic Archaeon *Methanosarcina acetivorans* Is Responsible for Hydroxyarchaeol Biosynthesis.” Biochemical and Biophysical Research Communications 466: 186–191. 10.1016/j.bbrc.2015.09.001. 26361140

[emi70213-bib-0049] Nauhaus, K. , A. Boetius , M. Krüger , and F. Widdel . 2002. “In Vitro Demonstration of Anaerobic Oxidation of Methane Coupled to Sulphate Reduction in Sediment From a Marine Gas Hydrate Area.” Environmental Microbiology 4: 296–305. 10.1046/j.1462-2920.2002.00299.x. 12080959

[emi70213-bib-0048] Nauhaus, K. , M. Albrecht , M. Elvert , A. Boetius , and F. Widdel . 2007. “In Vitro Cell Growth of Marine Archaeal‐Bacterial Consortia During Anaerobic Oxidation of Methane With Sulfate.” Environmental Microbiology 9: 187–196. 10.1111/j.1462-2920.2006.01127.x. 17227423

[emi70213-bib-0050] Niemann, H. , and M. Elvert . 2008. “Diagnostic Lipid Biomarker and Stable Carbon Isotope Signatures of Microbial Communities Mediating the Anaerobic Oxidation of Methane With Sulphate.” Organic Geochemistry 39: 1668–1677. 10.1016/j.orggeochem.2007.11.003.

[emi70213-bib-0051] Niemann, H. , T. Lösekann , D. de Beer , et al. 2006. “Novel Microbial Communities of the Haakon Mosby Mud Volcano and Their Role as a Methane Sink.” Nature 443: 854–858. 10.1038/nature05227. 17051217

[emi70213-bib-0052] Omoregie, E. O. , V. Mastalerz , G. De Lange , et al. 2008. “Biogeochemistry and Community Composition of Iron‐ and Sulfur‐Precipitating Microbial Mats at the Chefren Mud Volcano (Nile Deep Sea fan, Eastern Mediterranean).” Applied and Environmental Microbiology 74: 3198–3215. 10.1128/AEM.01751-07. 18378658 PMC2394935

[emi70213-bib-0053] Orphan, V. J. , C. H. House , K.‐U. Hinrichs , K. D. McKeegan , and E. F. DeLong . 2001. “Methane‐Consuming Archaea Revealed by Directly Coupled Isotopic and Phylogenetic Analysis.” Science 293: 484–487. 10.1126/science.1061338. 11463914

[emi70213-bib-0054] Orphan, V. J. , C. H. House , K.‐U. Hinrichs , K. D. McKeegan , and E. F. DeLong . 2002. “Multiple Archaeal Groups Mediate Methane Oxidation in Anoxic Cold Seep Sediments.” Proceedings of the National Academy of Sciences 99: 7663–7668. 10.1073/pnas.072210299. PMC124316 12032340

[emi70213-bib-0055] Osorio‐Rodriguez, D. , K. S. Metcalfe , S. E. McGlynn , et al. 2023. “Microbially Induced Precipitation of Silica by Anaerobic Methane‐Oxidizing Consortia and Implications for Microbial Fossil Preservation.” Proceedings of the National Academy of Sciences of the United States of America 120: e2302156120. 10.1073/pnas.2302156120. 38079551 PMC10743459

[emi70213-bib-0056] Pancost, R. D. , J. S. Sinninghe Damsté , S. de Lint , M. J. E. C. van der Maarel , and J. C. Gottschal . 2000. “Biomarker Evidence for Widespread Anaerobic Methane Oxidation in Mediterranean Sediments by a Consortium of Methanogenic Archaea and Bacteria.” Applied and Environmental Microbiology 66: 1126–1132. 10698781 10.1128/aem.66.3.1126-1132.2000 PMC91952

[emi70213-bib-0057] Pernthaler, A. , A. E. Dekas , C. T. Brown , S. K. Goffredi , T. Embaye , and V. J. Orphan . 2008. “Diverse Syntrophic Partnerships From Deep‐Sea Methane Vents Revealed by Direct Cell Capture and Metagenomics.” Proceedings of the National Academy of Sciences 105: 7052–7057. 10.1073/pnas.0711303105. PMC2383945 18467493

[emi70213-bib-0059] Pernthaler, A. , J. Pernthaler , and R. Amann . 2002. “Fluorescence in Situ Hybridization and Catalyzed Reporter Deposition for the Identification of Marine Bacteria.” Applied and Environmental Microbiology 68: 3094–3101. 10.1128/AEM.68.6.3094-3101.2002. 12039771 PMC123953

[emi70213-bib-0058] Pernthaler, J. , F.‐O. Glöckner , W. Schönhuber , and R. Amann . 2001. “Fluorescence in Situ Hybridization (FISH) With rRNA‐Targeted Oligonucleotide Probes.” In Methods in Microbiology, 207–226. Elsevier. 10.1016/S0580-9517(01)30046-6.

[emi70213-bib-0060] Pop Ristova, P. , F. Wenzhöfer , A. Ramette , J. Felden , and A. Boetius . 2015. “Spatial Scales of Bacterial Community Diversity at Cold Seeps (Eastern Mediterranean Sea).” ISME Journal 9: 1306–1318. 10.1038/ismej.2014.217. 25500510 PMC4438319

[emi70213-bib-0061] Rao, A. , and A. J. M. Driessen . 2024. “Unraveling the Multiplicity of Geranylgeranyl Reductases in Archaea: Potential Roles in Saturation of Terpenoids.” Extremophiles 28: 14. 10.1007/s00792-023-01330-2. 38280122 PMC10821996

[emi70213-bib-0062] Ravenschlag, K. , K. Sahm , C. Knoblauch , B. B. Jørgensen , and R. Amann . 2000. “Community Structure, Cellular rRNA Content, and Activity of Sulfate‐Reducing Bacteria in Marine Arctic Sediments.” Applied and Environmental Microbiology 66: 3592–3602. 10.1128/AEM.66.8.3592-3602.2000. 10919825 PMC92189

[emi70213-bib-0063] Reeburgh, W. S. 2007. “Oceanic Methane Biogeochemistry.” Chemical Reviews 107: 486–513. 10.1021/cr050362v. 17261072

[emi70213-bib-0064] RStudio Team . 2024. RStudio: Integrated Development Environment for R.

[emi70213-bib-0065] Ruff, S. E. , J. F. Biddle , A. P. Teske , K. Knittel , A. Boetius , and A. Ramette . 2015. “Global Dispersion and Local Diversification of the Methane Seep Microbiome.” Proceedings of the National Academy of Sciences 112: 4015–4020. 10.1073/pnas.1421865112. PMC4386351 25775520

[emi70213-bib-0066] Sahonero‐Canavesi, D. X. , M. F. Siliakus , A. Abdala Asbun , et al. 2022. “Disentangling the Lipid Divide: Identification of Key Enzymes for the Biosynthesis of Membrane‐Spanning and Ether Lipids in Bacteria.” Science Advances 8: eabq8652. 10.1126/sciadv.abq8652. 36525503 PMC13159169

[emi70213-bib-0068] Salvador‐Castell, M. , B. Demé , P. Oger , and J. Peters . 2020. “Lipid Phase Separation Induced by the Apolar Polyisoprenoid Squalane Demonstrates Its Role in Membrane Domain Formation in Archaeal Membranes.” Langmuir 36: 7375–7382. 10.1021/acs.langmuir.0c00901. 32515591

[emi70213-bib-0069] Salvador‐Castell, M. , M. Golub , N. Erwin , et al. 2021. “Characterisation of a Synthetic Archeal Membrane Reveals a Possible New Adaptation Route to Extreme Conditions.” Communications Biology 4: 653. 10.1038/s42003-021-02178-y. 34079059 PMC8172549

[emi70213-bib-0070] Salvador‐Castell, M. , M. Tourte , and P. M. Oger . 2019. “In Search for the Membrane Regulators of Archaea.” International Journal of Molecular Sciences 20: 4434. 10.3390/ijms20184434. 31505830 PMC6770870

[emi70213-bib-0067] Salvador‐Castell, M. , N. Brooks , R. Winter , J. Peters , and P. Oger . 2021. “Non‐Polar Lipids as Regulators of Membrane Properties in Archaeal Lipid Bilayer Mimics.” International Journal of Molecular Sciences 22: 6087. 10.3390/ijms22116087. 34200063 PMC8200183

[emi70213-bib-0071] Santana‐Molina, C. , E. Rivas‐Marin , A. M. Rojas , and D. P. Devos . 2020. “Origin and Evolution of Polycyclic Triterpene Synthesis.” Molecular Biology and Evolution 37: 1925–1941. 10.1093/molbev/msaa054. 32125435 PMC7306690

[emi70213-bib-0072] Scheller, S. , H. Yu , G. L. Chadwick , S. E. McGlynn , and V. J. Orphan . 2016. “Artificial Electron Acceptors Decouple Archaeal Methane Oxidation From Sulfate Reduction.” Science 351: 703–707. 10.1126/science.aad7154. 26912857

[emi70213-bib-0073] Skennerton, C. T. , K. Chourey , R. Iyer , R. L. Hettich , G. W. Tyson , and V. J. Orphan . 2017. “Methane‐Fueled Syntrophy Through Extracellular Electron Transfer: Uncovering the Genomic Traits Conserved Within Diverse Bacterial Partners of Anaerobic Methanotrophic Archaea.” MBio 8: e00530‐17. 10.1128/mBio.00530-17. 28765215 PMC5539420

[emi70213-bib-0074] Sturt, H. F. , R. E. Summons , K. Smith , M. Elvert , and K.‐U. Hinrichs . 2004. “Intact Polar Membrane Lipids in Prokaryotes and Sediments Deciphered by High‐Performance Liquid Chromatography/Electrospray Ionization Multistage Mass Spectrometry—New Biomarkers for Biogeochemistry and Microbial Ecology.” Rapid Communications in Mass Spectrometry 18: 617–628. 10.1002/rcm.1378. 15052572

[emi70213-bib-0075] Thauer, R. K. 2011. “Anaerobic Oxidation of Methane With Sulfate: On the Reversibility of the Reactions That Are Catalyzed by Enzymes Also Involved in Methanogenesis From CO_2_ .” Current Opinion in Microbiology 14: 292–299. 10.1016/j.mib.2011.03.003. 21489863

[emi70213-bib-0076] Treude, T. , A. Boetius , K. Knittel , K. Wallmann , and B. Barker Jørgensen . 2003. “Anaerobic Oxidation of Methane Above Gas Hydrates at Hydrate Ridge, NE Pacific Ocean.” Marine Ecology Progress Series 264: 1–14. 10.3354/meps264001.

[emi70213-bib-0077] Treude, T. , M. Krüger , A. Boetius , and B. B. Jørgensen . 2005. “Environmental Control on Anaerobic Oxidation of Methane in the Gassy Sediments of Eckernförde Bay (German Baltic).” Limnology and Oceanography 50: 1771–1786. 10.4319/lo.2005.50.6.1771.

[emi70213-bib-0078] Treude, T. , V. Orphan , K. Knittel , A. Gieseke , C. H. House , and A. Boetius . 2007. “Consumption of Methane and CO2 by Methanotrophic Microbial Mats From Gas Seeps of the Anoxic Black Sea.” Applied and Environmental Microbiology 73: 2271–2283. 10.1128/AEM.02685-06. 17277205 PMC1855681

[emi70213-bib-0079] Villanueva, L. , J. S. S. Damsté , and S. Schouten . 2014. “A Re‐Evaluation of the Archaeal Membrane Lipid Biosynthetic Pathway.” Nature Reviews. Microbiology 12: 438–448. 10.1038/nrmicro3260. 24801941

[emi70213-bib-0080] Wang, F.‐P. , Y. Zhang , Y. Chen , et al. 2014. “Methanotrophic Archaea Possessing Diverging Methane‐Oxidizing and Electron‐Transporting Pathways.” ISME Journal 8: 1069–1078. 10.1038/ismej.2013.212. 24335827 PMC3996691

[emi70213-bib-0086] Wegener, G. , H. Niemann , M. Elvert , K.‐U. Hinrichs , and A. Boetius . 2008. “Assimilation of Methane and Inorganic Carbon by Microbial Communities Mediating the Anaerobic Oxidation of Methane.” Environmental Microbiology 10: 2287–2298. 10.1111/j.1462-2920.2008.01653.x. 18498367

[emi70213-bib-0081] Wegener, G. , M. Bausch , T. Holler , et al. 2012. “Assessing Sub‐Seafloor Microbial Activity by Combined Stable Isotope Probing With Deuterated Water and 13C‐Bicarbonate.” Environmental Microbiology 14: 1517–1527. 10.1111/j.1462-2920.2012.02739.x. 22498240

[emi70213-bib-0082] Wegener, G. , M. Y. Kellermann , and M. Elvert . 2016. “Tracking Activity and Function of Microorganisms by Stable Isotope Probing of Membrane Lipids.” Current Opinion in Biotechnology 41: 43–52. 10.1016/j.copbio.2016.04.022. 27179643

[emi70213-bib-0085] Wegener, G. , R. Laso‐Pérez , V. J. Orphan , and A. Boetius . 2022. “Anaerobic Degradation of Alkanes by Marine Archaea.” Annual Review of Microbiology 76: 553–577. 10.1146/annurev-micro-111021-045911. 35917471

[emi70213-bib-0083] Wegener, G. , V. Krukenberg , D. Riedel , H. E. Tegetmeyer , and A. Boetius . 2015. “Intercellular Wiring Enables Electron Transfer Between Methanotrophic Archaea and Bacteria.” Nature 526: 587–590. 10.1038/nature15733. 26490622

[emi70213-bib-0084] Wegener, G. , V. Krukenberg , S. E. Ruff , M. Y. Kellermann , and K. Knittel . 2016. “Metabolic Capabilities of Microorganisms Involved in and Associated With the Anaerobic Oxidation of Methane.” Frontiers in Microbiology 7: 46. 10.3389/fmicb.2016.00046. 26870011 PMC4736303

[emi70213-bib-0087] Whiticar, M. J. 1999. “Carbon and Hydrogen Isotope Systematics of Bacterial Formation and Oxidation of Methane.” Chemical Geology 161: 291–314. 10.1016/S0009-2541(99)00092-3.

[emi70213-bib-0088] Wickham, H. , M. Averick , J. Bryan , et al. 2019. “Welcome to the Tidyverse.” Journal of Open Source Software 4, no. 43: 1686. 10.21105/joss.01686.

[emi70213-bib-0089] Wishnak, S. 2022. “New Frontiers in Ocean Exploration: The Ocean Exploration Trust, NOAA Ocean Exploration, and Schmidt Ocean Institute 2021 Field Season.” Oceanography 35: 1–78. 10.5670/oceanog.2022.supplement.01.

[emi70213-bib-0090] Zander, Y. 2024. yaza11/pyGC_FID_processing: alpha. 10.5281/ZENODO.13961282.

[emi70213-bib-0091] Zhu, Q.‐Z. , G. Wegener , K.‐U. Hinrichs , and M. Elvert . 2022. “Activity of Ancillary Heterotrophic Community Members in Anaerobic Methane‐Oxidizing Cultures.” Frontiers in Microbiology 13: 912299. 10.3389/fmicb.2022.912299. 35722308 PMC9201399

